# DLQI scores in vitiligo: reliability and validity of the Persian version

**DOI:** 10.1186/1471-5945-4-8

**Published:** 2004-08-04

**Authors:** Shahin Aghaei, Manouchehr Sodaifi, Peyman Jafari, Nazila Mazharinia, Andrew Y Finlay

**Affiliations:** 1 Department of Dermatology, Shiraz University of Medical Sciences, Shiraz, Iran; 2 Department of Biostatistics, Shiraz University of Medical Sciences, Shiraz, Iran; 3 Burn Hospital, Shiraz University of Medical Sciences, Shiraz, Iran; 4 Department of Dermatology, University of Wales College of Medicine, Cardiff CF4 4XN, UK

## Abstract

**Background:**

The objective of this study was to translate and to test the reliability and validity of the 10-item Dermatology Life Quality Index (DLQI) questionnaire in Iranian patients with vitiligo.

**Methods:**

Using a standard "forward-backward" translation procedure, the English language version of the questionnaire was translated into Persian (the Iranian official language) by two bilinguals. Seventy patients with vitiligo attending the Department of Dermatology, Saadi Hospital, Shiraz, Iran, were enrolled in this study.

The reliability and internal consistency of the questionnaire were assessed by Cronbach's alpha coefficient and Spearman's correlation, respectively. Validity was performed using convergent validity.

**Results:**

In all, seventy people entered into the study. The mean age of respondents was 28.3 (SD = 11.09) years. Scores on the DLQI ranged from 0 to 24 (mean ± SD, 7.05 ± 5.13). Reliability analysis showed satisfactory result (Cronbach's α coefficient = 0.77). There were no statistically significant differences between daily activity (DA) and personal relationship (PR) scale mean scores in generalized versus focal-segmental involvement in sufferers (P = 0.056, P = 0.053, respectively). There were also strong differences between the mean scores of the PR (personal relationship) scale with the involvement of covered only and covered/uncovered areas (P= 0.016) that was statistically significant in the second group.

**Conclusions:**

The study findings showed that the Persian version of the DLQI questionnaire has a good structural characteristic and is a reliable and valid instrument that can be used for measuring the effects of vitiligo on quality of life.

## Background

Vitiligo is an important skin disease having major impact on the quality of life of patients suffering from vitiligo. Appearance of skin can condition an individual self-image, and any pathological alteration can have psychological consequences [1,2]. Many vitiligo patients feel distressed and stigmatized by their condition. These patients often develop negative feeling about it, which are reinforced by their experiences over a number of years. Most patients of vitiligo report feelings of embarrassment, which can lead to a low self-esteem and social isolation [3]. The Dermatology Life Quality Index questionnaire is designed for use in adults, i.e. patients over the 16. It is self-explanatory and can be simply handed to the patient who is asked to fill it in without the need for detailed explanation. It is usually completed in one to two minutes. The questions were classified to 6 headings items: symptoms and feelings (questions 1 and 2), daily activities (questions 3 and 4), leisure (questions 5 and 6), and personal relationships (questions 8 and 9) each item with maximum score 6; work and school (question 7), and treatment (question10) each item with maximum score 3 [4].

The DLQI is calculated by summing the score of each question resulting in a maximum of 30 and a minimum of 0. The higher the score, the more quality of life is impaired. The DLQI can also be expressed as a percentage of the maximum possible score of 30.

The scores for each of these sections can also be expressed as a percentage of either 6 or 3.

Since the DLQI is a brief, simple, easy to complete, and its application in research settings as a screening tool is well documented, it was decided to translate the DLQI into Persian (the Iranian official language) and to examine reliability and validity of this questionnaire in an Iranian population with vitiligo.

## Methods

The standard "forward-backward" procedure was applied to translate the questionnaire from English into Persian. Two independent bilinguals translated the items and two others translated the response categories and after cultural adaptation, a provisional version was provided. Subsequently, it was back translated into English and then the final version was provided. The cultural adaptation was done by the translation of the "partner" to the "spouse" in Persian language and adding of it in question 8 and 9, respectively.

The final draft of the Persian version was administered to a sample of patients with vitiligo that referred to Department of Dermatology, Saadi Hospital, Shiraz, Iran. There were no restrictions on patient selection with regard to extension of lesions. The patients were introduced to the subject of this study and informed about the personal nature of the questionnaire, and all those who gave consent were given the DLQI questionnaire to complete. The questionnaires were completed by the patients whom were referred to our clinic for psoralen and UVA (PUVA) therapy. The patients were categorized by extension of lesions into covered only (vitiligo patches are covered by cloths) and covered/uncovered involvement and by severity of disease to focal-segmental (focal is defined as a single or a few depigmented macules that are located in a discrete area, segmental is the unilateral localization of one or more macules to one area of the body) [5], which in this study were settled in one category, and generalized involvement groups (widespread distribution of numerous macules over the integument in a random pattern) [6]. Age of patients, marital status and the number of treatment sessions were recorded. Responses on the DLQI were scored according to the guidelines of Finlay and Khan [4]. All statistical analyses were carried out using the Statistical Package for the Social Sciences (SPSS 11.0 for Windows). To test the reliability, the internal consistency of the questionnaire was assessed by Cronbach's alpha coefficient and alpha equal to or greater than 0.70 was considered satisfactory [7].

Validity was performed using convergent validity to demonstrate the extent to which the DLQI correlated with global quality of life. Construct validity was checked by factor analysis.

## Results

Seventy patients aged 18 to 68 (mean ± SD, 28.3 ± 11.09) years completed the questionnaire. We did not have any incomplete questionnaires; therefore we included all questionnaires in our study.

Scores on the DLQI ranged from 0 to 24(mean ± SD, 7.05 ± 5.13). The reliability of the questionnaire was obtained by Cronbach's alpha coefficient (α = 0.77). There were no statistically significant differences between the sex, item scores and mean DLQI score. Table 1 shows the results of item convergent validity tests. The scaling success rates were 100% for convergent validity of each scale.

**Table 1:** Item scaling tests: convergent validity for DLQI scales

**Scale**	**No. of items per scale**	**Convergent validity (range of correlation)**	**Scaling Success^1^**	**Scaling Success Rate^2^**	**Internal consistency (Cronbach's α)**
**SF**	2	0.69–0.78	2/2	100	0.70
**DA**	2	0.71–0.78	2/2	100	0.76
**L**	2	0.67–0.88	2/2	100	0.71
**WS**	1	1.00	1/1	100	1.00
**PR**	2	0.50–0.99	2/2	100	0.79
**T**	1	1.00	1/1	100	1.00

**SF **(***Symptoms and feelings***), **DA **(***Daily activities***), **L **(***Leisure***), **WS **(***Work and School***),**PR **(***Personal relationships)***, **T **(***Treatment***) 1- Number of correlation between items and hypothesized scale corrected for overlap ≥ 0.4/ total number of convergent validity tests. 2- Scaling success rate is the previous column as a percentage.

Cronbach's α coefficient by gender, marital status, severity, and extension of disease are shown in Table 2.

**Table 2:** Cronbach's coefficient by gender, marital status, severity, and extension of disease

**Variable**	**Cronbach's coefficient (n^1^)**
**Gender**	
Male	0.60 (27)
Female	0.80 (43)
**Marital status**	
Single	0.79 (42)
Married	0.75 (28)
**Severity**	
Focal/Segmental	0.58 (18)
Generalized	0.79 (52)
**Extension**	
Covered/Uncovered	0.78 (54)
Covered	0.67 (16)

^1 ^The number of patients in each category.

There were no statistically significant differences between item and mean DLQI scores of males versus females and married versus single cases.

Cronbach's α reliability coefficients ranged from 0.69 to 0.78 for symptoms and feelings (SF) scale, 0.71 to 0.78 for daily activities (DA) scale, 0.67 to 0.88 for leisure (L) scale, and 0.50 to 0.99 for personal relationships (PR) scale. The reliability coefficient for work and school (WS) scale was equal to treatment (T) scale that was 0.100. Table 2 shows comparison of Cronbach's α in each scale separately. The DA scale was found to have a strong association with gender (female scores were greater than male ones).

The L scale was found to have significant relationship with the severity of the disease (generalized versus focal/segmental) (P value = 0.018).

The DA and PR scales, also had no statistical association with severity factor (P = 0.056 and P = 0.053, respectively).

The PR scale had strong correlation with the type of the extension of lesions (covered only versus covered/uncovered type) (P value = 0.016).

There was no association between the numbers of treatment sessions with the type of disease (generalized versus focal/segmental).

The number of treatment sessions and mean DLQI score was found to have a positive correlation coefficient (P value = 0.02, r = 0.28) but this correlation was statistically significant in the generalized type only (P value = 0.008, r = 0.37).

Spearman's correlation coefficient of severity of the disease with questions 5, 6, and 8 were 0.25, 0.24, and 0.26 respectively. For question 8 and the extension of disease and also for question 1 and the stage of disease it was equal to 0.26.

The result in question 4 and gender status was statistically significant (0.008) there was no statistically significance correlation between the age and each of questions.

The result of question 9 and marital status was statistically significant (P = 0.002) and it was higher in married patients. The range of the paired correlations between the items was 0.17–0.68.

Factor analysis is performed to determine the Persian version is a two-dimensional measure including social and psychological parameters (Table 3).

**Table 3:** Factor loadings (rotated) ^1 ^of two-factor solution

**DLQI items**	**Social factor**	**Psychological factor**
**Q 1**	.086	**.485**
**Q 2**	**.545**	.305
**Q 3**	**.535**	.374
**Q 4**	**.470**	.285
**Q 5**	**.564**	.473
**Q 6**	.190	**.468**
**Q 7**	.088	**.619**
**Q 8**	**.813**	.186
**Q 9**	**.681**	-.195
**Q 10**	.099	**.500**

^1 ^Varimax

## Discussion

The DLQI questionnaire is a well-known instrument for measuring dermatological distress and has been translated into a variety of languages [8,9] and [10].

The translation process set by the international quality of life assessment (IQOLA) project was built on lessons from cross-cultural psychology and other health survey projects to develop protocols for translating, validating, and norming health status questionnaires, entails forward translation by at least two translators who were native speakers of the target language, rating of translation equality by two other bilinguals, and back translation by two translators who were native speakers of American-English or British-English [11]. Because native English speakers were unavailable, we did not fully adhere to this strategy. Two independent Iranian health professionals translated the items and subsequently, it was back translated into English by two others and then the final version was provided.

Vitiligo is an acquired depigmentation disorder of great cosmetic importance affecting 1–4% of the world's population. The disease has a major impact on quality of life of patients, many of whom feel stigmatized by their condition [12].

Porter et al. studied the effect of vitiligo on sexual relationships and found that embarrassment during sexual relationships was especially frequent for men with vitiligo [13]. Salzer and Schallreuter reported that 75% found their disfigurement moderately or severely intolerable [14].

Weiss et al compared the difficulties faced by patients with vitiligo with those with leprosy in India [15]. There may be a relationship between stress and the development of vitiligo. Al-Abadie et al. indicated that psychological stress increases levels of neuroendocrine hormones, affects the immune system and alters the level of neuropeptides, which may be the initial steps in pathogenesis of vitiligo [16].

In general, the finding of this study indicated that mental health in vitiligo patients is poor and it is strongly associated with their quality of life. Since the patients with higher DLQI scores responded less favorably to a given therapeutic modality [12], improving quality of life in this group becomes very important task.Severity (generalized versus focal/segmental) and extension of lesions on covered only or covered/uncovered areas has an effect on quality of life of patients. 

This study reports data from a validation study of the 10-item DLQI questionnaire in Iran. In general the findings showed promising results and were comparable with other research finding throughout the world [12]. The two-dimensional Persian version of DLQI questionnaire assessed the social and psychological difficulties as other studies [12]. There was no relationship of DLQI score with gender, which is consistent with the study of Parsad et al. [12].

The mean DLQI score in this study was 7.05 that is lower than that obtained by Finlay and Khan (mean 7.3) [4] and Parsad et al. (mean 10.67) [12], and it is higher than Kent and Al-Abadie's study (mean 4.82) [17].

Reliability was associated by internal consistency of the questionnaire reporting Cronbach's alpha coefficient and validity was examined by convergent validity showed satisfactory results (α = 0.77). Cronbach's α was < 0.7 for males, focal/segmental, and covered vitiligo that may be related to small sample size and cultural differences.

The Persian version of the DLQI questionnaire proved to be acceptable to patients and it is worth nothing that occasionally the questionnaire was administered by a trained nurse in face-to-face interviews. However this was done in illiterate patients and some ones indicated that some questions were difficult to answer, especially question 8. Perhaps this was the reason why a weaker correlation was found for this item with its corresponding subscale.

## Conclusions

The study finding showed that the Persian version of the DLQI questionnaire has a good structured characteristic and is a reliable and valid instrument that can be used for measuring the effects of the vitiligo on quality of life. Especially, the reliability of this questionnaire was high in females and patients with generalized involvement, because of the great cosmetic importance in these groups.

## Competing interests

None declared.

## Abbreviations

DLQI: Dermatology Life Quality Index

SD: standard deviation

IQOLA: international quality of life assessment

## Pre-publication history

The pre-publication history for this paper can be accessed here:


